# **African Physician Migration to High-Income Nations: Diverse Motives to Emigrate ("We Are not Florence Nightingale") or Stay in Africa ("There Is No Place Like Home")** Comment on "Doctor Retention: A Cross-sectional Study of How Ireland Has Been Losing the Battle"

**DOI:** 10.34172/ijhpm.2020.219

**Published:** 2020-11-02

**Authors:** Akhenaten Siankam Tankwanchi, Amy Hagopian, Sten H. Vermund

**Affiliations:** ^1^Department of Health Services, School of Public Health, University of Washington, Seattle, WA, USA.; ^2^Department of Global Health, School of Public Health, University of Washington, Seattle, WA, USA.; ^3^Office of the Dean, Yale School of Public Health, Yale University, New Haven, CT, USA.

**Keywords:** Global Labor Mobility, Health Workforce Migration, Health Personnel Retention, Right to Health, Freedom of Movement, Sub-Saharan Africa

## Abstract

Research in assessing the global and asymmetric flows of health workers in general, and international medical graduates in particular, is fraught with controversy. The complex goal of improving health status of the citizens of home nations while ensuring the right of health workers to migrate generates policy discussions and decisions that often are not adequately informed by evidence. In times of global public health crises like the current coronavirus disease 2019 (COVID-19) global pandemic, the need for equitable distribution and adequate training of health workers globally becomes even more pressing. Brugha et al report suboptimal training and working conditions among Irish and foreign medical doctors practicing in Ireland, while predicting large-scale outward migration. We comment on health personnel migration and retention based on our own experience in this area of research. Drawing from our examination of medical migration dynamics from sub-Saharan Africa, we argue for greater consideration of health workforce retention in research and policy related to resource-limited settings. The right to health suggests the need to retain healthcare providers whose education was typically subsidized by the home nation. The right to migrate may conflict with the right to health. Hence, a deeper understanding is needed as to healthcare worker motives based on interactions of psychosocial processes, economic and material determinants, and quality of work environments.

Commenting on the need to regulate more rigorously the entry of foreign-trained doctors into the physician workforce of the United States, the late Dr. Fitzhugh Mullan explained: “A doctor in almost every country in the world is a product of the taxpayers or the tax base of that country.”^1^ Countries invest considerable resources in the medical and nursing education of their citizens with expectations that they will deliver essential healthcare services to the local population after completing their training. When the medical, dental, nursing, pharmacy, lab and other health sector graduates leave their native countries to settle abroad for practice, expectations for public return on investments are not realized, health workforce planning is compromised, and healthcare delivery is undermined.^2^ Estimated costs of physician (and presumably nurse) migration from lower to higher income venues are based on both loss of human capital and also excess mortality associated with loss of expertise.^3^One can characterize this controversial problem as a battle between the “right to health”^4^ (which countries invest in by supporting training programs) vs. the right to emigrate protected by the 1948 Universal Declaration of Human Rights. While the loss of high-value human resources is especially problematic for low- and middle-income countries (LMICs) with weak health systems and critical health workforce shortages,^2^ high-income countries can also suffer from large-scale migration of their health workers as noted in Brugha and colleagues’ recent study of doctor retention in Ireland.^5^

In a national sample of nearly 1150 junior doctors practicing in Irish hospitals, Brugha et al found half (52%) of respondents intended to leave Ireland either before, during, or after completing their specialty training. Although most of these prospective emigrants reported they plan to return to Ireland later to practice, the authors acknowledged that intention to return may diminish with time if the main drivers of emigration are not addressed. One caveat is that intention to migrate does not always translate into actual migration and, further, actual migration does not always translate to the ability to practice the profession in the new location. However, such “brain waste” may largely apply to international medical graduates from non-English speaking LMICs immigrating to leading Anglophone destination countries.^6^

Notwithstanding Ireland’s long-established medical migration culture and its role as staging ground for international medical graduates contemplating onward migration,^7^ migration is a contextual, opportunistic, and dynamic phenomenon. Would-be migrants’ options for realizing their emigration and international career plans will depend to a large extent on their qualifications, the legal framework governing migration, the licensure requirements and labor market in destination countries,^8^ and the prevailing global public health context and risks as evidenced by the travel bans and disruptions created by the ongoing pandemic of coronavirus disease 2019 (COVID-19). It is notable the US Department of State has special facilitating guidance for foreign medical professionals trying to get to the United States during the COVID-19 surge, and for those already in the country.^9^ Since the authors collected their data prior to the COVID-19 outbreak, Brugha et al could not examine the potential effect of the novel coronavirus disease on respondents’ current attitudes toward migration and post-COVID-19 emigration intentions and trends. Given the seriousness of the pandemic in both the United States and the United Kingdom, we are doubtful the combined proportion (36%) of respondents who elected these two countries as their intended destinations will stick to their choices should they decide to emigrate in the near future.

Several of the main findings reported by Brugha et al, are not new and can be generalizable across many countries, especially their findings related to the English-speaking leading destination countries. As observers of physician emigration trends from the World Health Organization (WHO) Region of greatest need,^10-12^ we were interested to learn more about Sub-Saharan African doctors participating in this Irish study. Of the 20% (n = 206) non-European Union respondents, how many were nationals of an African country? What proportion of these African doctors intended to stay and practice in Ireland after specialty training? What proportions contemplated forward or circular migrations and why? The aggregated nature of the data analysis does not enable us to learn answers to these questions.

 One of the key findings from Brugha and colleagues’ study is that “bullying” pervades many Irish healthcare settings, and this may be an added factor in outward migration. Although the authors did not define bullying, given the prevailing context of global acknowledgments of systemic racism against individuals of African descent, we would have liked to know what proportion, if any, of African respondents reported experiencing bullying, and to what extent were their experiences more negative or traumatic than those of non-African émigrés? We hope the authors will delve further into these questions.

One may have sympathy for migrating Irish doctors who long to escape suboptimal working conditions some have reported in Ireland. But few observers of global health disparities and inequities will dispute the general fact that health service delivery and working conditions are far worse in resource-limited settings of LMICs. Often, low salary is cited as a prime driver of physician and nurse migration from LMICs to higher income nations, but the issue is surely more complex. One factor of emigration could be one’s disposition and intentions from the very start of medical or nursing school. A second is whether one has actually grown up (or trained) in an area of need, such as an LMIC rural area, in which case emigration is less likely.^13^

Understanding what motivates or enables some professionals to leave and others to stay under similar circumstances is a perennial question in skilled/professional migration research that cannot be fully examined through quantitative methods alone. We have explored the determinants of medical migration and health personnel retention in Sub-Saharan African countries using both quantitative data from the American Medical Association Masterfile and qualitative data from in-depth interviews with both African-based medical practitioners and US-based African medical graduates.^10,11^ To highlight some of the complexities and nuances of migration, we illustrate our rendering with two examples from Ghanaian identical twin physicians we interviewed in the context of our previous work.^10,14^ The first set of twins graduated in tandem from the faculty of medicine of Kwame Nkrumah University of Science and Technology in 1990, but made diametrically opposed migration decisions. One brother migrated to the United States more than 20 years ago (and reports he does not regret his decision), while his twin decided to stay and practice medicine in Ghana. In explaining the circumstances of his decision against migration, the latter repeatedly stated: “There is no place like home.”^14^ The second set of twin sisters graduated from the University of Ghana Medical School in the early 2000s and moved together to the United States a year later. One of the sisters, then a critical care medicine fellow in a Washington, DC residency program, asserted her freedom to emigrate while explaining the instrumental motivation for pursuing a career in medicine:

*“Ghana has a need for physicians, and I understand that. But at the same time, I also appreciate the fact that there is freedom. We live in a global village. If I want to go and live in Timbuktu, that’s where I go. If I want to go and live in China, that’s where I go. You can’t hold anybody back. … And physicians are not charity people. When they are selecting us to go to medical school, we are choosing our career. We are not Florence Nightingale. We like to do medicine, for the same reason that somebody else wants to be a lawyer.”*

We have argued elsewhere that migration can be construed as a liberatory process which involves both notions of “freedom to” and “freedom from.”^10^ Hence, affirming one’s freedom to emigrate in order to expand one’s opportunities, implicitly affirms one’s desire to free oneself from the local sources of structural violence and stunted opportunity that compromise one’s personal wellbeing and professional satisfaction.^14^ Within the context of her working conditions in a tertiary referral hospital of her native Ghana, the above émigré expressed her frustration as follows:

*“The biggest facility where I trained, Korle-Bu Teaching Hospital, which is like the national hospital in Ghana, is a dirty hospital; so many of its facilities are broken down, and nothing is being done to fix it. … The system is such that physicians are handicapped in carrying out their duties. It is like working in a jungle. … People come with chest pain, but you cannot do cardiac enzyme in the night; you cannot do it on the weekends. And these are things that are time sensitive. You can’t wait 24 hours to do some of these tests. So, for me practicing medicine in Ghana is frustrating.”* ^14^

This account of dire conditions of service in Ghana’s public hospitals was corroborated by many interviewees, including colleagues who trained and worked alongside the aforementioned émigré in the same Accra-based teaching hospital and under the same substandard conditions but who did not emigrate despite also having opportunities to do so.^10,14^ One such local doctor in her mid-1930s, an age by which 85% of African medical migrants to the United States had already moved,^11^ explained her commitment and attachment to her homeland in this manner:

*“I am staying in my country because, the truth is that I love Ghana. I believe that no matter what you do, and where you go, and who you become, you will never be a first-class citizen in anybody else’s country but your own. … I am not against travel. I like traveling. But I don’t think that I would like to go and live in another person’s country. Because no matter what I do (I may have a Green Card, a British passport, or whatever), I will always be a second-class citizen. But in Ghana, wherever I work, wherever I go, this is my home. For me, I love this country, and that is why I stayed. … I have really good friends who think like I do. We believe that Ghana is our home, and we stay in Ghana, and we work in Ghana. And then if you need to go for a holiday or travel, you save up some money and then you go. But, by all means we come back to our home, and we will be here.”* ^14^

 The contrasting attitudes and responses of these two classmates to the same adverse working conditions suggest intangible factors operating outside the health systems and impervious to the economic determinants and material incentives that reportedly draw and trap many physicians to high-income countries. These factors should be explored in research and policy discussions of health personnel retention.

Public medical school admissions policies in LMICs might be reconsidered to assess dispositional factors likely to predict prospective students’ post-graduation inclination to practice in the nation that invested in their medical training. Research suggests certain non-academic attributes such as individualism and altruism can predict medical and nursing students’ intention to emigrate or to stay and serve rural and remote areas in LMICs.^13^ While the quality and extent of such evidence should be systematically evaluated before making any lasting policy, as community psychologist, public health professor, and pediatrician respectively, we believe it important to consider such dispositional attributes in the recruitment of trainees in caring professions like medicine and nursing. Further, social and behavioral theories and research on place attachment, place identity, and sense of community provide insight into the psychosocial processes that bond individuals with their native communities and places.^15^ To enable the production of health cadres most likely to stay or return from abroad to practice in their countries, we recommend health personnel retention policy informed by insights into community attachment processes and candidate dispositional attributes. It is likely such policies will also serve the rural and most impoverished urban communities in home countries

## Ethical issues

 Not applicable.

## Competing interests

 AST reports personal fees from World Health Organization (WHO), outside the submitted work.

## Authors’ contributions

 AST wrote the first draft of the commentary. AH and SHV revised the draft critically for important intellectual content. All three authors approved the final submission.

## Authors’ affiliations

^1^Department of Health Services, School of Public Health, University of Washington, Seattle, WA, USA. ^2^Department of Global Health, School of Public Health, University of Washington, Seattle, WA, USA. ^3^Office of the Dean, Yale School of Public Health, Yale University, New Haven, CT, USA.
